# CT maxillary sinus evaluation-A retrospective cohort study

**DOI:** 10.4317/medoral.20513

**Published:** 2015-04-10

**Authors:** Inês Guerra-Pereira, Paula Vaz, Ricardo Faria-Almeida, Ana-Cristina Braga, António Felino

**Affiliations:** 1 DDS, Invited Assistant, Department of Oral Surgery, Faculty of Dentistry, Oporto University, Portugal; 2 DDS, PhD, Auxiliary Professor, Department of Orofacial Genetics, Faculty of Dentistry, Oporto University; 3 DDS, MdS, PhD, Associate Professor, Department of Oral Surgery, Faculty of Dentistry, Oporto University; 4 Assistent Professor, PhD, Department of Production and Systems Engineering, University of Minho, Braga, Portugal; 5 DDS, PhD, Professor, Department of Oral Surgery, Faculty of Dentistry, Oporto University

## Abstract

**Background:**

Proximity of the dental roots to the sinus floor makes dental disease a probable cause of maxillary sinusitis. The aim of this study was to find out if maxillary sinus pathologic changes were more prevalent in patients with dental disease and to evaluate the performance of computed tomography (CT) in analyzing and detecting apical periodontitis and other odontogenic causes on the maxillary sinusitis etiology in a Portuguese Caucasian population.

**Material and Methods:**

Retrospective cohort study. The total sample of 504 patients and their CT was included in this study. The patients were from a private dental clinic, specializing in oral surgery, where the first complaint was not directly related to sinus disease, but with dental pathology. For each patient, the etiological factors of maxillary sinusitis and the imaging CT findings were analyzed. All the axial, coronal and sagittal CT slices were evaluated and general data were registered. The latter was selected based on the maxillary sinus CT published literature.

**Results:**

32.40% of patients presented normal sinus (without any etiological factor associated), 29.00% showed presence of etiological and imaging findings in the maxillary sinus, 20.60% had only imaging changes in the maxillary sinus and 18.00% of patients presented only etiological factors and no change in the maxillary sinus.

**Conclusions:**

Radiological imaging is an important tool for establishing the diagnosis of maxillary sinus pathology. These results indicate that the CT scan should be an excellent tool for complement the odontogenic sinusitis diagnosis.

**Key words:** Maxillary sinusitis/etiology, odontogenic, computed tomography, maxillary sinus.

## Introduction

The pathological extension of dental disease into the maxillary sinus is a condition first described by Maloney, in 1968, as maxillary sinusitis of dental origin (1).

The inferior maxillary sinus wall is a curved structure formed by the lower third of the medial wall and the buccoalveolar wall (2). The maxillary sinus floor is consisted by the alveolar process of the maxilla (3). The adult maxillary sinus is variable in its extension. In about half of the general population, the maxillary sinus floor extends between adjacent teeth or individual roots, creating elevations in the antral surface, commonly referred to as ‘hillocks’(3,4). The roots of the maxillary premolar, molar and occasionally of the canine teeth may project into the maxillary sinus(2,4). In 2012, a study that evaluated 332 maxillary molars in Cone Beam Computed Tomography (CBCT) demonstrated that: the buccal roots of the maxillary teeth were closer to the maxillary sinus, the mesial buccal root (MB) of the second molar was the closest to the maxillary sinus and the MB root of the first molar was closer to the vestibular cortical (2). So, probably this anatomical design may explain the odontogenic source and development of an inflammatory process into the maxillary sinus. Also, this close relationship is, probably, responsible for the 37.00%-40.60% maxillary sinusitis cases of odontogenic origin, described by some authors (5-8).

Before the 1970s, it was believed that the odontogenic sinusitis accounted only for 10.00% to 12.00% of maxillary sinusitis cases (1). Despite this, it has been suggested that the incidence of sinusitis of dental etiology is increasing (9). In 1982, Lindahl *et al*. identified an odontogenic maxillary sinusitis prevalence of 47.00%, based on the clinical and radiographic evaluation of 62 Swedish patients (6). A study carried out in 411 Romanian patients by Albu and Baciut (2010) based on a dental examination and on a computed tomography (CT) analysis, reported a maxillary sinusitis prevalence of 25.00% (10).

The CT imaging allows 3D observation and clear visualisation of the inflammatory changes present in the nasal and paranasal sinus mucosa. Therefore this is the reason for the choice of this method as a valuable tool for assessing the pathologic status of nasal and paranasal sinuses (11). According to some authors, the CT also allows to determine the existence of a possible dental focus (responsible for sinus pathology), as well as the study of the maxillary sinus condition(7,12,13). Concerning possible dental foci, some authors also pointed out the chronic oral antral fistula (OAF), the presence of foreign bodies (dental fillings, teeth roots or broken instruments), of periapical granulomas and small inflammatory cysts of the molars and bicuspids, of large odontogenic cysts occupying total or subtotal space of the maxillary sinus (5,14).

Mélen highlighted the difficulty on diagnosing maxillary odontogenic sinusitis, inherent to the slow progress of the dental infections and to the minor symptoms before an exacerbation occurs (8). So, the maxillary odontogenic sinusitis pathogenesis is still not clearly understood and there is a lack of consensus concerning its clinical features, treatment, and prevention. The aim of this study was to find out if maxillary sinus pathologic changes were more common in patients with dental disease and to evaluate the performance of computed tomography (CT) in analyzing and detecting apical periodontitis and other odontogenic causes on the maxillary sinusitis etiology in Portuguese Caucasian population. Indeed, the authors aimed to answer two questions: 1- maxillary sinus pathologic changes observed on CT images are more prevalent in Portuguese Caucasian patients with dental disease? 2- Is the computed tomography (CT) a good tool for analyzing and detecting apical periodontitis and other odontogenic causes in cases of maxillary sinusitis etiology, in a Portuguese Caucasian population?.

## Material and Methods

The present investigation represents a retrospective cohort study based on computed tomography (CT) scans analysis of 504 Portuguese dental clinic patients, for the period between 1990 and 2013. The patients were from a private dental clinic, specializing in oral surgery, where the first complaint was not directly related to sinus disease, but with dental pathology. Written informed consent was obtained from all patients included in this study. Institutional review board approval (of Ethical Commission of Faculty of Dentistry of Oporto University) was obtained by, and appropriate measures were taken to safeguard patient privacy.

The performance of CT was not in any of the cases purposely for this study, but according to the following: pre-implant study, study of impacted maxillary teeth, suspected lesion / cyst, ankylosis of maxillary teeth, suspected foreign body, injury endodontic, oral-antral fistula, orthodontic planning, trauma, fractures, or chronic pain.

From a total of 947 patients, the inclusion and exclusion criteria were applied. The inclusion criteria included adult Caucasian Portuguese patients who had at least one CT scan and patients with both, left and right, maxillary sinus exposure. The exclusion criteria contemplated: patients with a CT scan but younger than 18 years old (62 excluded); patients with CT scan, from whom it was impossible to collect all the information (53 excluded); immune-compromised patients with CT scan (1 excluded due to leukemia); patients with CT scan only regarding the jaw (295 excluded); patients with CT scan without sufficient slices or poor quality image to analyse the maxillary sinus (27 excluded); patients with CT scan that did not allow to analyse the maxillary teeth (5 excluded).

The total number of CT’s obtained was divided into four groups based on the classification of the study of Maillet and Abrahams (7,15).

Group IA (patients with etiological factors of dental origin and imaging changes in maxillary sinus) - density of soft tissue mass or mucosal thickening more than 2 mm within the sinus, and presence of one of the following criteria: decayed tooth, tooth restoration faulty, extraction site with mucosal thickening.

Group IB (patients with etiological factors of dental origin and without imaging changes in maxillary sinus) – absence of mucosal thickening or uniform mucosal thickening less than 2 mm. Adjacent teeth should: evidence signs of carious lesions, be decayed, have exposed pulp, be restored, be extracted and have imaging of apical periodontitis (presence of potential etiologic odontogenic factors).

Group IIA (patients without etiological factors of dental origin and with imaging changes in maxillary sinus) - density of soft tissue mass within the sinus, being fulfilled the following criteria: healthy teeth, coronal restoration and /or endodontic good quality, absence of periapical lesion, tooth extracted intact or healed alveoli and thickening of the mucosa not limited to any tooth.

Group IIB (patients without etiological factors of dental origin and without imaging changes in maxillary sinus) – absence of mucosal thickening or uniform mucosal thickening less than 2 mm defective, being fulfilled the following criteria: healthy teeth, coronal restoration and /or endodontic good quality, absence of periapical lesion, tooth extracted intact or healed alveoli and thickening of the mucosa not limited to any tooth (absence of potential etiologic odontogenic factors).

Two operators (with more than 20 years of experience in oral surgery) performed independently the CT scan analysis. All the axial, coronal and sagittal slices were observed and general data, such as age, gender and place date of CT acquisition were collected.

According to the current literature the potential etiological factors for maxillary sinusitis of odontogenic origin were defined and evaluated on CT: dental caries, periodontal disease, apical periodontitis, endodontics, iatrogenic, implants, cysts, foreign bodies, ectopic teeth, oro-antral fistula and impacted teeth associated with maxillary sinus (5).

Radio graphically, periodontal disease was been identified as a radio lucent bag together with adjacent bone resorption and loss of gingival attachment on the periodontal ligament (15). The periapical radio lucency was considered associated with the apical region of the root if the width of the radio lucency exceed at least twice the width of the periodontal ligament space (16). When pulp disease was present in a tooth (whose apex approached the maxillary sinus floor), the periapical region of the tooth involved could be presented with a radiolucency, by a radiographic loss of lamina dura of the involved tooth or even by the leakage of endodontic obturation material (17). The presence of the implants was considered pathological when it was over 4 mm inserted into the sinus space (18).

Only the adjacent teeth to the maxillary sinus were evaluated (dental and periodontal assessments). Edentulism (from teeth 13 or 23) and teeth protruding into the maxillary sinus were also evaluated.

The pathologic maxillary sinus changes for evaluation included: mucosal thickening (12), presence of air-fluid (19), opacification and bone loss (13), according to literature (20).

As for the mucosa thickening, we used the criterion of Maillet *et al*. (7) and Lu *et al*. (12) , in which the thickening of the mucosa from 2 mm was considered pathological. This was considered only when the mucosa thickening location was related with the presence of dental origin pathology. The presence of air-fluid in the sinus was assessed based on the presence of a fluid opacification, noted in the axial plane of the image, according with Bomeli criterion (19).

The reliability of radiographic measurement was assessed measuring the index of conformity of intra-and between-observer. The latter, usually performed to analyse the agreement between two observation intervals (intra-observer) and between observers (inter- observers), inter-observer reliability was done by statistical measure of reliability Cohen’s kappa. The variables in the study were randomly selected from 10% of the sample (82 maxillary sinus, 41 individuals) and reassessed in a second time by the same observer. All the collected data were recorded and analysed in the IBM® SPSS® Statistics version 22 (New York, USA). The latter aimed to characterize the sample and to determine if the dental imaging findings were associated with maxillary sinus changes.

The decision rule consisted of detect statistical evidence if the *p* value of the test was less than 0.05.

## Results

**Figure 1:**
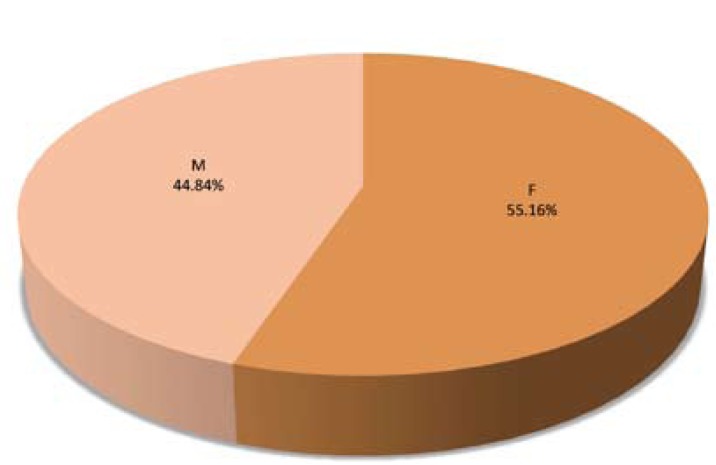
Sample gender distribution.

CT scans observed allowed the selection of 504 CT of Portuguese Caucasian individuals for the sample, of whom 55.20% (278) were female (F) and 44.80% (226) were male (M) (Fig. 1).

The mean patient age was 39.29 years with a standard deviation of 14.32 years (ranged 18-82 years). As for the dental analysis results, we could find that 9.90% of the patients were toothless/missing teeth on the right side and 9.70% of them were toothless on the left side. Among the dentate patients, 43.80% had maxillary teeth in contact with the right maxillary sinus and 47.30% had the same similar contact in the left maxillary sinus.

To assess the reliability of radiological measurements, the degree of agreement beyond what would be expected, solely by chance and usually, ranges from 0-1 (although negative numbers are possible) where a large number means greater reliability, values near or less than zero suggest that the agreement is attributable to chance. Therefore, results were evaluated in terms of the coefficient of Kendal tau as shown in  Table 1, which shows excellent agreement for all observed variables.

**Table 1:**
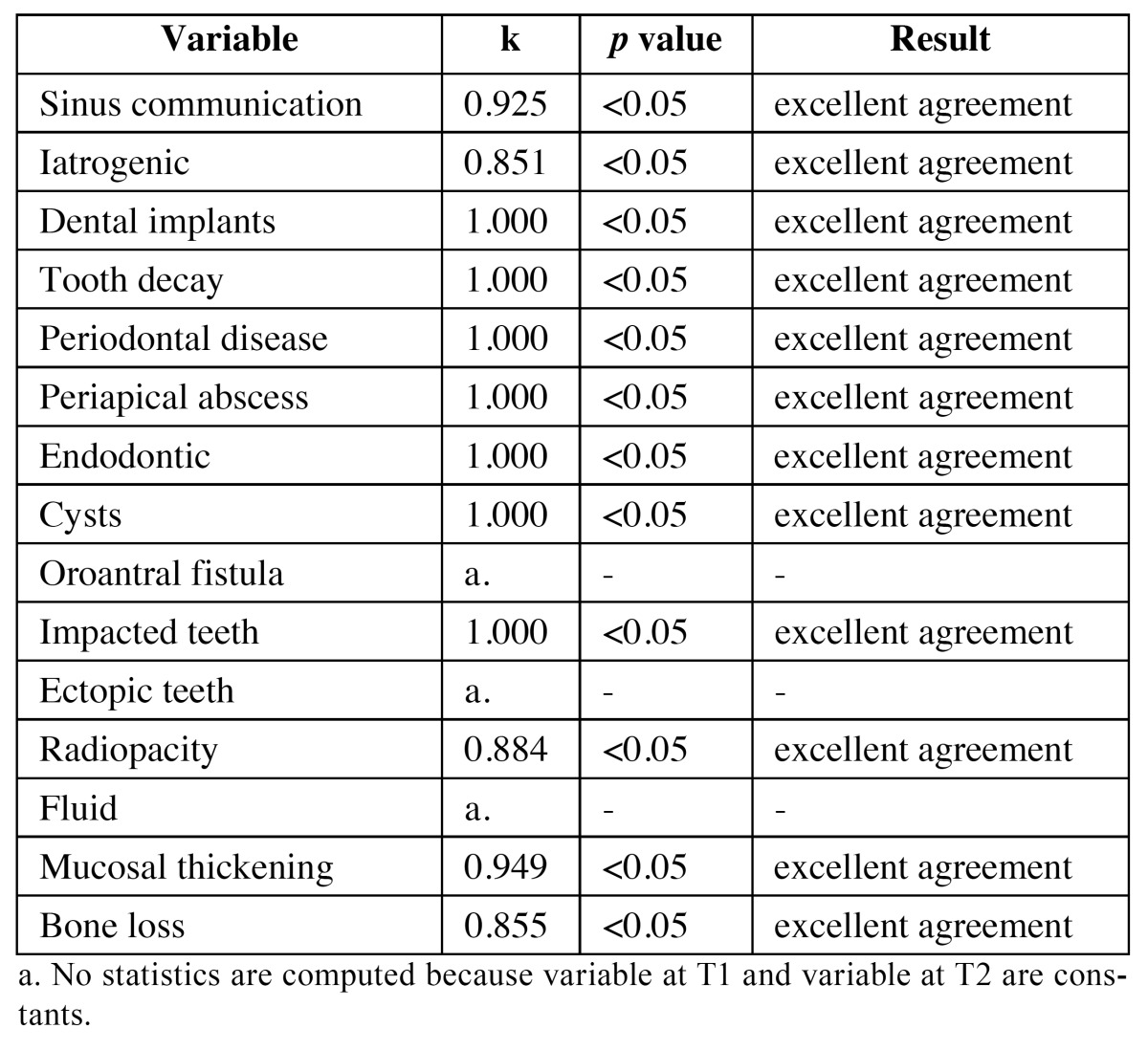
Results of interobserverreliabilityevaluated in terms of the coefficient of Kendal tau.

Due to this is a clinic specializing in oral surgery, the reason for performing CT was 41.80% pathology, the further study of the lesion previously identified in panoramic radiography. The remaining CTs were performed for reasons other than pathology (study pre-implant or orthodontic planning). According to imaging evidence and regarding the adopted diagnosis methodology, it was possible to verify that: 32.40% of the patients presented maxillary sinus without pathologic changes and without any etiological factor associated (Group IIB); 29.00% showed presence of etiological and imaging changes in the maxillary sinus (Group IA); 20.60% had only imaging changes in the maxillary sinus (Group IIA) and 18.00% of the patients presented only etiological factors and had absence of changes in the maxillary sinus (Group IB).

Potential odontogenic etiologic factors of pathologic changes in maxillary sinus, that were more often present in the group IA, included the endodontic treatment (25.15%), apical lesions (20.43%), presence of cysts (14.93%) and periodontal disease (8.25%).

To evaluate the association between etiological factors and changes in the maxillary sinus, an independence chi-squared test was performed ( Table 2).

**Table 2:**
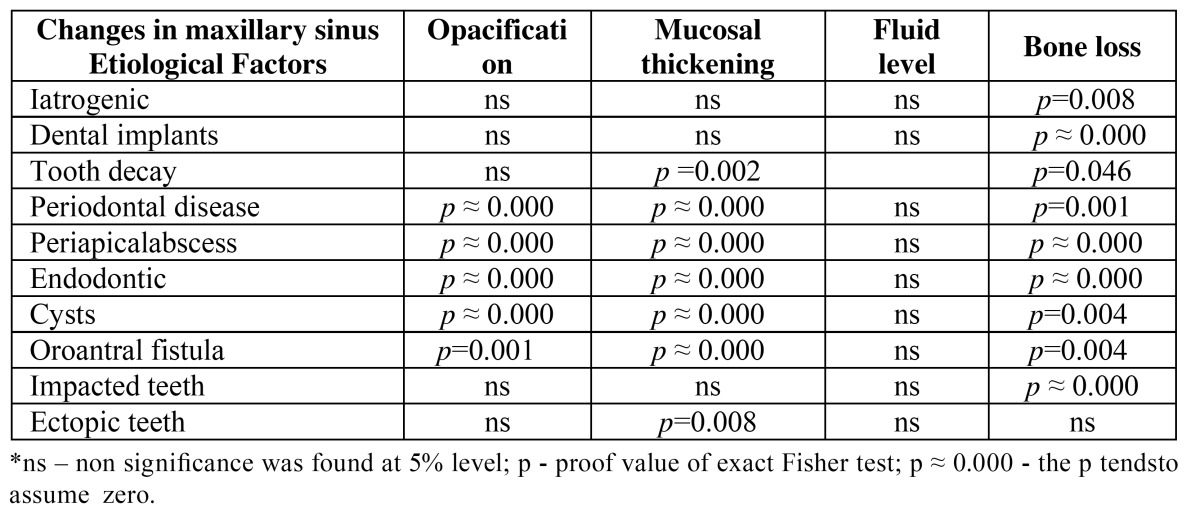
Results of Fisher exact test.

Regarding the communication between the maxillary teeth and the maxillary sinus, it was possible to conclude that there was a statistically significant association between proximity of the maxillary sinus and the presence of opacification, mucosa thickening and bone loss ( Table 2).

**Figure 2:**
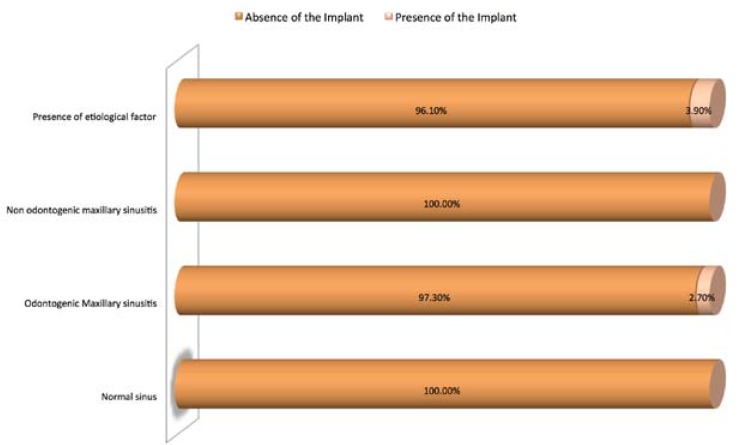
Distribution of diagnosis according to the presence of dental implants in the maxillary sinus.

The independence chi-square test revealed a statistically significant association between the group’s classification and the presence of dental implants (χ2 = 18.126, df = 3, *p* ≈ 0.000 <0.05). That is to say that the presence of etiological factor for odontogenic sinusitis and the presence of pathologic maxillary sinus imaging tends to be associated with the presence of dental implants in the maxillary sinus (Fig. 2).

## Discussion

The diagnosis criteria adopted in our study comprised a modification of the classification performed by different authors: Abrahams and Glassberg (15), Maillet (7) and the pathologic imaging changes described by Pinheiro (20). The classification of Abrahams and Glassberg, which was developed by radiologists, did not evaluate carious lesions or endodontic status of the involved tooth (15). In the study of Maillet, odontogenic maxillary sinusitis was identified as a localized thickening of the mucous membrane of the maxillary sinus. The modified classification system, adopted in our study, allowed comprehensive analysis of sinusitis of an odontogenic origin, analysing individually each one of the potential etiologic factors of the pathology and imaging changes present in maxillary sinus.

Radio graphically, the normal maxillary sinus has a shape that is inconsistent, with many loci and lobulations. Since it is filled with air, the sinus is radiolucent, but it has clearly defined margins (1). In the case of a diseased sinus, a clinician may observe clouding (opacification), mucosal thickening, and/or accumulation of fluid (20,21).

Previous studies have reported that the prevalence of maxillary sinusitis is higher in women (5,13,22), but in our study there was no significant difference in the prevalence between genders. Such difference could be related to specific characteristics of each study population.

As for the result patient average age found in our sample, it was 39.29±14.32years. The latter is someway in accordance with some studies (5,8,23) and in discordance with the published in other Works (24).

Mélen *et al*. reported that only 2 of the 99 patients of their sample, that had maxillary sinusitis, were under the age of 30 (8). The meta-analysis of Arias-Irimia *et al*. showed that the fourth decade was the most frequently affected age group (5). As for the study of Lee, it was reported that the average age of the patients with maxillary sinusitis was 42.9 years (23). Also, the study of Pokorny *et al*. reported the presence of only 5 patients younger than 30 years old (16.00%) and presented a balanced middle age distribution (13).

On the other hand, we have the Longhini *et al*. study. This revealed the presence of odontogenic maxillary sinusitis in 21 patients and reported the following results for age and gender: 11 patients were female and 10 were male, with a mean age of 53 years (range 21-70 years) (24).

In our study, the prevalence of changes in maxillary sinus evaluated by CT scan together with an odontogenic etiological factor (Group IA), suggesting sinusitis of odontogenic origin (29.00%), proved to be higher than what was reported in the classic literature (1,25). However, recent studies have also reported even higher results, related to the presence of odontogenic sinusitis, than the presented by our work (9,13,14,19,26).

So, Obayashi *et al*. found that 71.30 % of cases with dental infection were associated with changes in the maxillary sinus. In this study the periapical pathology was first diagnosed and was followed by radiographic examination of the sinuses (26).

Bomeli *et al*. revealed that cases of acute maxillary sinusitis, with CT imaging signs, more likely to be related with a dental cause, accounted for 86.00% of his study simple (19). An apparent increase in the incidence of odontogenic sinusitis (with no cases reported in 2004 compared with the 10 cases presented in 2009) seems to have occurred over the last decade in the UK (9). Moreover, the study of Maillet also presented a higher odontogenic sinusitis incidence of 51.80% (70 in 135 cases) (7).

The study of Pokorny and Tataryn (2013) reported again a higher value for the cases of odontogenic maxillary sinusitis present on imaging CT findings (13). So, these authors showed in a total of eighteen sample cases (55.00%), the presence of odontogenic maxillary sinusitis with an obvious CT image of periapical abscess, three of them with associated failing root canal therapy (13). Also, three sample cases (9.00%) had advanced periodontal disease with no clinical finding of endodontic infection. Moreover, the periodontal described lesions were clinically correlated and found to create effective oroantral fistula and their appearance on CT imaging was obvious and similar to periapical abscess. Four cases (12.00%) had failing root canal therapy, without obvious active abscess formation, and six cases (18.00%) had lack of bone. The latter was characterized by absence of the bony partition between the root apices and the sinus mucosa, or tooth roots that protrude through the maxillary sinus cortical floor and directly contacted the sinus mucosa. Added to this, the Pokorny and Tataryn study reported that approximately two-thirds (64.00%) of the maxillary sinusitis of odontogenic sinusitis cases showed evident periradicular infection on CT (13).

This difference, related to the imaging analysis (suggesting odontogenic maxillary sinusitis) found in our results could be attributed to the differences associated with characteristics of the population study, as for example, the age or even the ethnic origin. These differences may also be related with the different diagnostic techniques and equipment used by the compared studies.

The most often etiologic factors related to development of pathological changes in maxillary sinus found in our research were: endodontic treatment, apical lesions, cysts, and periodontal disease. These results are in accordance with some studies and also in discordance with other ones, as following described.

Periodontal disease, as an etiological factor of sinusitis, was reported a long time ago. In 1943, after performing studies on corpses, Bauer proved the existence of direct dissemination of a buccal sepsis to the maxillary sinus (25).

Abrahams *et al*. observed that sinusitis incidence on patients with periodontal disease was double to the present on patients without periodontal disease (15).

Important to note that, according to Arias-Irimia meta-analysis and colleagues (5) and recent studies on this topic, periodontal disease also did not appeared as the main etiological factor of odontogenic maxillary sinusitis. This can be explained, probably, due to the fact that, in the recent years, the population experienced an improvement of the oral hygiene and due to the periodontal disease preventive techniques implemented.

Lopatin *et al*. reported that, at the time of surgery, foreign bodies were found in 21 sinuses, among them: tooth roots (in 11 cases), dental fillings (in 7 cases), and packs (in 3 cases). The latter were of iatrogenic origin and had been inserted in the sinus as a result of dentists’ actions, during their attempts to pack the fistula after tooth extraction (14).

As for the meta-analysis of Arias-Irimia *et al*., it was described the iatrogenia as the most frequent cause of odontogenic maxillary sinusitis (55.97%) (5).

Other etiologic factors reported included: periodontitis (40.38%); odontogenic cysts (6.66%); oroantral fistulas, remaining rooted and iatrogenia after tooth extraction (47.56%); nonspecific foreign bodies (19.72%). Poor dental implants or their migration to the maxillary sinus, 0.92% of all cases, were also included under a iatrogenic source (5).

Lee *et al*. found that dental implant-related complications were the most common cause of maxillary sinusitis, found in 10 (37.00%) of the 27 patients. Dental extraction-related complications were the second most common cause, found in 8 of the 27 patients (29.60%). A dentigerous cyst was seen in 3 of the patients of the Lee study sample (11.10%). In the latter, a radicular cyst, dental caries, and a supernumerary tooth were the least common causes, with each found in 2 (7.40%) of the 27 patients (21). It is known that the incidence of sinusitis associated with dental implants is very low, despite the high frequency of dental implants. However, this incidence has been gradually increasing, according to Lee *et al*. (23).

Lee study also revealed that bony erosion of the involved maxillary sinus was presenting 12 cases (44.40%) (23). In cases of lack of bone, this normal anatomic condition exposes the tooth roots directly to maxillary sinus mucosa. The infected dental roots, which protrude through the cortical sinus floor, are in direct contact with sinus mucosa, eliciting only inflammatory soft-tissue changes and imaging fluid level amendments. Another study showed that cortical bone in the floor of the sinus was disrupted in all cases, in which a dental etiologic factor was identified (7).

Dentists are, generally, unable to reliably detect dental infection causing maxillary sinusitis. The work of Longhini and Ferguson demonstrated that one-half of patients went to the dentist during their sinus disease, but only 1 in 7 (14.00%) was noted to have dental pathology on dental X-ray (24). Thus, dental pathology was missing in 86.00% of cases. This is similar to the reported by Mélen *et al*., that revealed that 56 in 99 (55.00%) of odontogenic maxillary sinusitis cases were missing on routine dental examination, including dental X-ray (8). Physicians should be mindful that a negative dental report does not exclude a dental etiology.

Possibly because our study population was recruited from a private dental clinic specialized in oral surgery, the patients were more likely to present severe dental pathology, indicating surgical intervention. Moreover, as one observer was an oral surgeon and the other one otolaryngologist, this could influence the identification of maxillary sinus pathologic findings and of dental pathology. The latter occurred maybe based on the higher experience and train of our observers when compared with other studies observers, which were radiologists (7,13,16,26).

Radiological imaging is an important tool for establishing the diagnosis. A CT is an excellent tool for diagnosing odontogenic sinusitis, because it can show the relationship between the odontogenic origin of the maxillary sinusitis, the sinus floor defect and the diseased tissues. According to the most retrospective studies in the literature that observed CT scans, they should be two observers experienced evaluating this, since it makes it more reliable validation of the observed data (11).

Preoperative evaluations of the patients, who suffer from previous symptoms of sinusitis or predisposing factors for it, are crucial to rule out structural drainage problems of the paranasal sinuses by intranasal observation and radiological examination. Identification of patients with an increased risk for developing odontogenic maxillary sinusitis should be considered a goal to achieve in the near future.
